# Transactivation of epidermal growth factor receptor through platelet-activating factor/receptor in ovarian cancer cells

**DOI:** 10.1186/s13046-014-0085-6

**Published:** 2014-09-28

**Authors:** Yi Yu, Mingxing Zhang, Xiaoyan Zhang, Qingqing Cai, Zhiling Zhu, Wei Jiang, Congjian Xu

**Affiliations:** Obstetrics and Gynecology Hospital, Fudan University, No.419 Fang-Xie Road, Shanghai, 200011 People’s Republic of China; Department of Obstetrics and Gynecology of Shanghai Medical School, Fudan University, No.138 Yi-Xueyuan Road, Shanghai, 200032 People’s Republic of China; Shanghai Key Laboratory of Female Reproductive Endocrine Related Diseases, No. 413 Zhao-Zhou Road, Shanghai, 200011 People’s Republic of China; Institute of Biomedical Sciences, Fudan University, Shanghai 200032, No.138 Yi-Xueyuan Road, Shanghai, 200032 People’s Republic of China

**Keywords:** Platelet-activating factor receptor, Epidermal growth factor receptor, Ovarian cancer cells, Transactivation

## Abstract

**Background:**

We previously identified platelet-activating factor receptor (PAFR) as being overexpressed in ovarian cancer and found that its ligand PAF evoked EGFR phosphorylation using the phospho-antibody microarray. Epidermal growth factor receptor (EGFR) are also overexpressed in ovarian cancer and contribute to the growth of ovarian cancer cells. Here, we investigated the mechanisms of crosstalk between PAFR and EGFR signaling in ovarian cancer cells to further determine whether the interaction between PAFR and EGFR synergistic contribute to the progression of ovarian cancer.

**Methods:**

Expression and localization of PAFR in several ovarian cancer cell lines were assessed by Western blot, realtime-PCR and immunofluorescence. The ovarian cancer cells were stimulated with PAF or PAF and in some experiments also pharmacological inhibitors. Phosphorylation of proteins in signaling pathways were measured by Western blot. HB-EGF concentrations of the supernatant from stimulated ovarian cancer cells were measured by enzyme-linked immunosorbent assay.

**Results:**

Our data show that PAF increases EGFR phosphorylation through PAFR in a time- and dose- dependent manner in SKOV-3 ovarian cancer cells. This transactivation is dependent on phospholipase C-β and intracellular calcium signaling. This pathway is also Src tyrosine kinase- and metalloproteinase- dependent. PAF triggers EGFR activation through the increased heparin-binding EGF-like growth factor (HB-EGF) release in metalloprotease-dependent manner. Several studies involving EGFR transactivation through G-protein coupled receptor (GPCR) have demonstrated EGFR-dependent increase in ERK1/2 phosphorylation. Yet in SKOV-3 cells, PAF treatment also increases ERK1/2 phosphorylation in a EGFR-independent manner.

**Conclusions:**

The results suggest that in SKOV-3 ovarian cancer cells, PAF transactivates EGFR and downstream ERK pathways, thus diversifying the GPCR-mediated signal. The crosstalk between PAFR and EGFR suggests a potentially important signaling linkage between inflammatory and growth factor signaling in ovarian cancer cells.

## Background

Platelet-activating factor (PAF), prostaglandins (PGs), and lysophosphatidic acid (LPA) are the three major phospholipid mediators implicated in many different biological pathways in inflammatory diseases and cancers [1-4]. Lipid mediators play a critical role in cancer initiation and progression. PAF induces diverse cellular effects through its specific receptor, PAFR, which belongs to the G-protein coupled receptor (GPCR) family and transduces cell signals via G-proteins and associated protein phosphorylation cascades [5-7]. Many types of cells, when challenged with PAF, showed the activation of tyrosine kinase [8] and protein phosphorylation [9-11].

The epidermal growth factor (EGF) receptor (EGFR) is upregulated in ovarian cancer, and increased expression is associated with reduced survival rate [12-15]. EGFR is emerging as an important therapeutic target for several epithelial tumors, including ovarian cancer. Yet, recent clinical trials targeting EGFR using cetuximab [16-18], matuzumab [19,20], gefitinib [21], and erlotinib [22,23] in epithelial ovarian cancer patients have shown only modest clinical responsiveness. The modest responses of EGFR blockade in response to the administration of monoclonal antibodies or tyrosine kinase inhibitors as single agents could be attributed to compensation through other signaling pathways.

It has been reported that different mechanisms might be involved in the crosstalk between GPCRs and EGFR [24]. Several GPCR ligands have been shown to activate the EGFR pathway contributing to carcinogenesis. PGE2 has been reported to activate the EGFR in hepatocarcinoma [25]. EGFR activation in response to GPCR ligands, including lysophosphatidic acid (LPA), has been reported in HNSCC cells [26]. Thus, the combined inhibition of EGFR and GPCR might enhance antitumor effects compared with single agents targeting EGFR alone. In our previous studies, we demonstrated PAFR gene and protein overexpression in ovarian cancer tissues and a series of ovarian cancer cell lines [27]. Phospho-antibody microarray analysis revealed increased EGFR, Src, FAK and Paxillin phosphorylation through PAF in OVCA429 and OVCA432 cells. We also showed that the combined PAFR and EGFR targeting synergistically inhibited the ovarian cancer *in vitro* and *in vivo* [28]. However, the mechanisms underlying EGFR phosphorylation through PAF/PAFR in human ovarian cancer have not yet been tested.

In the present study, we examined the SKOV-3, a well-characterized human serous ovarian cancer cell line with high levels of endogenous functional PAF-receptor expression, to characterize the interaction between the pathways mediated through PAFR and EGFR. The aim of this study was to determine whether PAF transactivates EGFR in ovarian cancer cells, examine the involvement of the PAFR in this process, and elucidate the intracellular signaling mechanisms required for transactivation. Activating growth factor receptors through PAF might be an important mechanism in mediating the downstream mitogenic effects of PAFR. This transactivation might reveal previously unknown associations between inflammatory and growth factor signaling, providing a better understanding of the relationship between inflammation and cancer.

## Materials and methods

### Cell culture and chemical reagents

The ovarian cancer cell lines SKOV-3, CAOV-3, OVCA433, RMUG-L and ES-2 (obtained from the Cell Bank of the Chinese Academy of Science, Shanghai, China) were maintained at 37°C in a humidified 5% CO_2_ atmosphere in RPMI-1640 medium supplemented with 10% fetal calf serum (Gibco, Invitrogen, Carlsbad, CA), 100 IU/ml penicillin G, and 100 mg/ml streptomycin sulfate (Sigma-Aldrich, St. Louis, MO). The cells were serum starved through incubation in serum-free medium for 12–24 hours before the start of the experiments. β-Acetyl-γ-O-alkyl-L-α-phosphatidylcholine (PAF), epidermal growth factor (EGF), WEB2086 (PAFR antagonist), AG1478 (EGFR inhibitor) and PP2 (Src inhibitor) were obtained from Sigma-Aldrich (St. Louis, MO). U73122 (PLC inhibitor), BAPTA-AM (calcium chelator), Thapsigargin (Ca^2+^-ATPase inhibitor), GF109203X (PKC inhibitor), and PMA (PKC activator) were obtained from Tocris (Bristol, UK). The rabbit polyclonal antibodies used in this study were directed against phospho/total-EGFR, phospho/total-ERK, and phospho/total-Src. All antibodies were purchased from Cell Signaling Technology (Boston, MA). The mouse monoclonal antibodies used in this study were directed against actin (Sigma, Missouri, USA).

### Western blot analysis

Cellular extracts were prepared in modified radioimmunoprecipitation assay (RIPA) buffer (50 mM Tris–HCl pH 7.4, 1% NP-40, 0.25% Na-deoxycholate, 150 mM NaCl, 1 mM EDTA, 1 mM PMSF, and protease inhibitor cocktail). The protein concentrations in the cellular extracts were measured using a Bio-Rad protein assay kit. The cellular extracts were subjected to SDS-PAGE, and the proteins were transferred onto PVDF membranes. After blocking for 1 h at room temperature in 5% BSA, the blots were incubated with the primary antibody at a 1:1000 dilution and incubated overnight at 4°C. Subsequently, the blots were washed three times and incubated for 1 h at room temperature with a 1:10000 dilution of secondary peroxidase-conjugated antibodies. After washing three times, the immunoreactive bands were detected using electrochemiluminescence (ECL).

### Quantitative real-time PCR

Total RNA was extracted using Trizol reagent (TaKaRa, Japan) and reverse transcribed using the PrimeScript RT-PCR kit (TaKaRa, Japan) according to the manufacturer’s instructions. Real-time PCR analyses were performed using SYBR *Premix Ex Taq* (TaKaRa, Japan) on a 7300 Real-time PCR system (Applied Biosystems, Inc. USA) at the recommended thermal cycling settings: one initial cycle at 95°C for 10 s, followed by 40 cycles at 95°C for 5 s and 60°C for 31 s. The following primer sequences were used for PAFR detection: sense, 5′- GGGGACCCCCATCTGCCTCA -3′ and antisense, 5′- GCGGGCAAAGACCCACAGCA -3′. The expression levels were normalized to the internal reference gene 18S rRNA (sense, 5′- GTAACCCGTTGAACCCCATT -3′ and antisense, 5′- CCATCAATCGGTAGTAGCG -3′) [29].

### Intracellular calcium measurement

Calcium mobilization was performed as described previously [30]. The SKOV3 cells were harvested with Cell Stripper (Mediatech, Herndon, VA, USA), washed twice with PBS and resuspended to 5 × 106 cells/ml in Hank’s balanced salt solution(140 mM NaCl, 5 mM KCI, 10 mM HEPES, pH7.4, 1 mM CaCI_2_, 1 mM MgCl_2_, 1 mg/ml glucose) containing 0.025% BSA. Subsequently, the cells were loaded with 3 μM Fura-2 acetoxymethyl ester derivative (Fura-2/AM) (Molecular Probes, Eugene, OR, USA) for 30 min at 37°C. The cells were washed once in Hank’s solution, and then resuspended in Hank’s at a concentration of 3 × 10^7^ cells/ml. These cells were subsequently stimulated with 100 nM PAF. The calcium flux was measured using excitation at 340 and 380 nm in a Tecan Infinite 200 pro series Microplate Reader (Tecan, Switzerland). When required, the cells were treated with WEB2086 for 1 hour in serum-free McCoy’s 5A medium prior to the experiment.

### HB-EGF assay

Aliquots of the supernatant from stimulated ovarian cancer cells were collected and the HB-EGF concentrations were measured using a specific enzyme immunoassay kit (Cayman Chemical, Ann Arbor, MI). The assay was performed according to the manufacturer’s instructions. HB-EGF production was evaluated in duplicates, and the concentrations were determined from a standard curve of HB-EGF. The sensitivity of the assay facilitated detection of up to 15 pg/ml. When necessary, the samples were diluted in the assay buffer.

### Immunocytochemistry

Following drug treatment, the cells were fixed with 100% methanol for 6 min at −20°C, washed with PBS and stored at 4°C until further use. The cells were permeabilized through incubation in PBS containing 0.3% Triton X-100 and 5% goat serum for 30 min. A polyclonal antibody against phospho-EGFR was used at a 1:100 dilution, and a secondary antibody FITC-conjugated goat anti-rabbit (Invitrogen, Carlsbad, CA), was used at a 1:200 dilution. The first antibody was incubated overnight at 4°C and the second antibody was incubated for 2 hours at RT. The images were captured using an Olympus DP 71 camera (Tokyo, Japan) at 400× magnification.

### Statistical analysis

All experiments were performed at least three times. The data are expressed as the means ± SD. Wherever appropriate, the data were also subjected to unpaired, two-tailed Student’s t-tests. The differences were considered significant at *P* < 0.05.

## Results

### PAF increases EGFR phosphorylation in SKOV-3 cells

**Figure 1:**
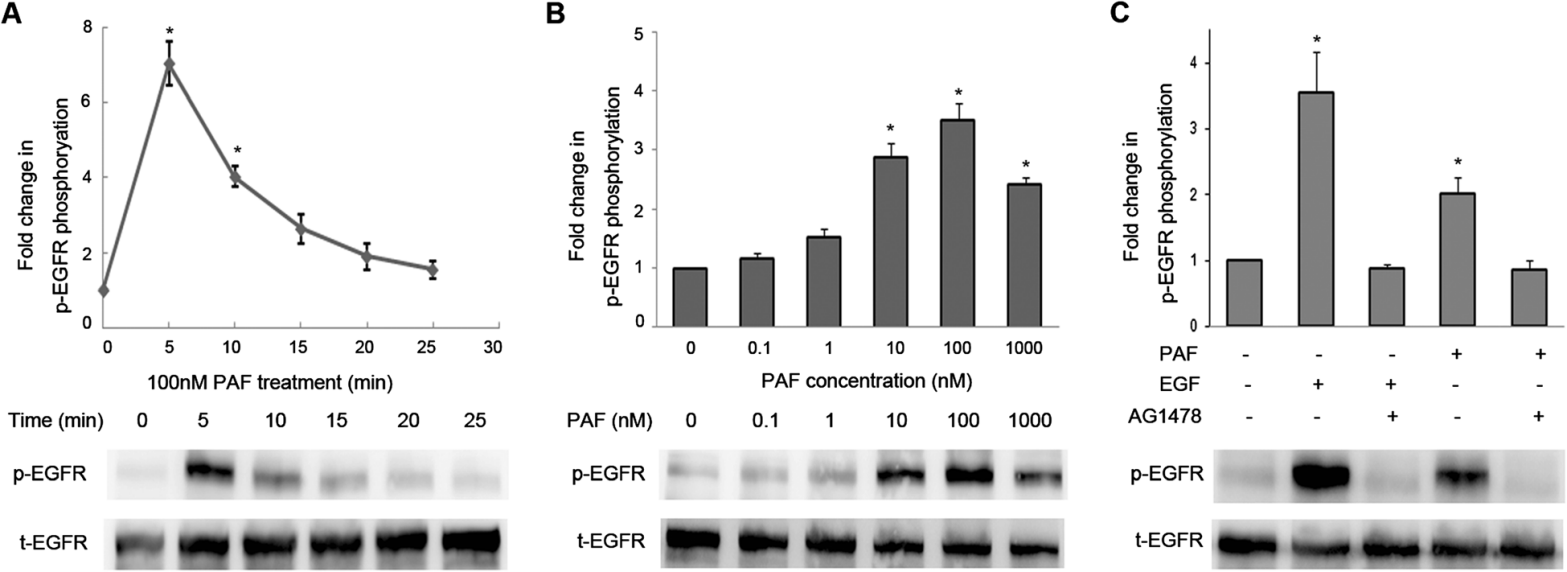
**Effects of platelet-activating factor (PAF) on epidermal growth factor receptor (EGFR) activation in SKOV-3 cells. (A)** SKOV-3 cells were treated with 100 nM PAF for 0, 5, 10, 15, 20, or 25 min. Following PAF treatment, the cells were lysed, and the lysates were evaluated by Western blotting. Data were normalized to total EGFR protein expression and are expressed as fold-change (average ± S.E.M.) in phospho-EGFR compared with vehicle-treated cells. Representative blots for phosphor/total-EGFR are shown. **(B)** SKOV-3 cells were treated with vehicle (0) or 0.1 to 1000 nM PAF for 5 min. Fold-phosphorylation data for immunoreactivity with antibodies against p-EGFR are shown in representative Western blots. **(C)** SKOV-3 cells were treated with AG1478 (20 μM) for 1 hour before stimulation with PAF (100 nM) or EGF (5 ng/ml) for 5 min. Cells were harvested and subjected to Western blotting. The data shown are representative of at least three independent experiments. Data were analyzed using Student’s *t*-test. * *p* < 0.05.

To determine the effects of PAF on EGFR transactivation in ovarian cancer cells, we used SKOV-3 for further investigations. In SKOV-3 cells, stimulation with PAF (100 nM) evoked EGFR phosphorylation in a time-dependent manner, with maximal activation at 5 min and a subsequent reduction to baseline after 25 min (Figure 1A). In addition, increasing concentrations of PAF increased EGFR phosphorylation in a dose-dependent manner, reaching maximum levels at 100 nM (Figure 1B). Furthermore, the phosphorylation of EGFR through PAF was inhibited using AG1478 (20 μM) (Figure 1C), a specific EGFR inhibitor [31], providing further support for the induction of EGFR transactivation through PAF in SKOV-3 cells.

### PAF-induced EGFR transactivation is dependent on the PAF-receptor

**Figure 2:**
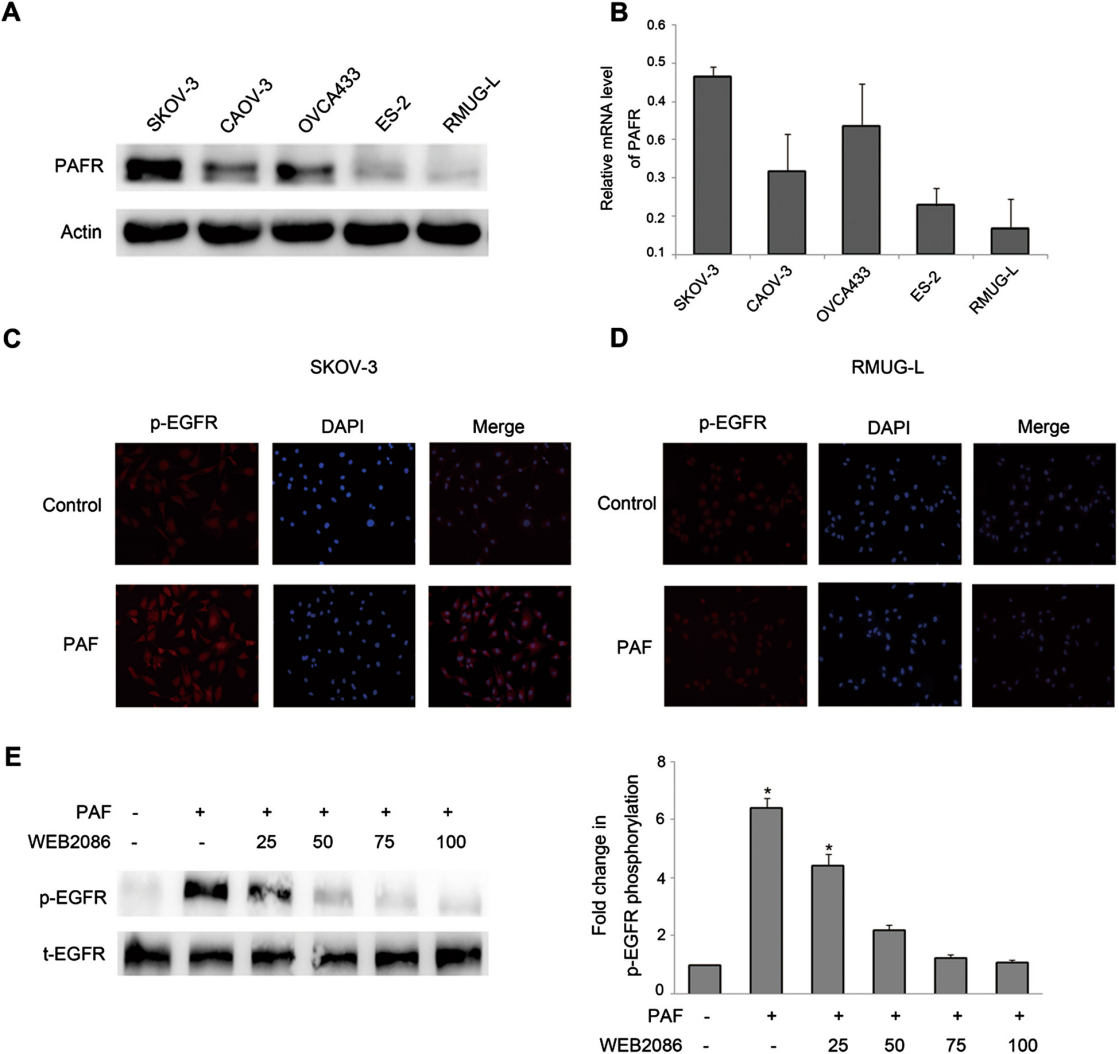
**PAF receptor is required for EGFR transactivation in ovarian cancer cells. (A)** Lysates from SKOV-3, CAOV-3, OVCA433, ES-2 and RMUG-L cells were subjected to SDS-PAGE, followed by immunoblotting for PAFR. Actin was used as loading control. **(B)** The relevant mRNA levels of PAFR in these five cell lines were tested by quantitative real-time PCR, and the data were normalized to 18S rRNA. For the immunofluorescence staining of phosphorylated EGFR in SKOV-3 **(C)** and RMUG-L **(D)** cells, after 10 min of incubation without any drug or with 100 nM PAF, the cells were labeled with a polyclonal antibody to phosphor-EGFR overnight, followed by incubation with fluorescent secondary antibody to phosphor-EGFR for 1 h and staining with DAPI for 10 min. **(E)** SKOV-3 cells were pretreated for 1 h with increasing concentrations of the PAFR antagonist WEB2086. The cells were then stimulated with PAF (100 nM) for 5 min before they were harvested and subjected to Western blotting. The data shown are representative of at least three independent experiments. Data were analyzed by Student’s *t*-test. * *p* < 0.05.

We next investigated whether the PAF-receptor (PAFR) is involved in the transactivation of EGFR in ovarian cancer cells. Western blotting and reverse-transcription polymerase chain reaction were used to examine five ovarian cancer cell lines. As shown in Figure 2A and B, PAFR expression was higher in SKOV-3, CAOV-3 and OVCA433 cell lines, whereas PAFR expression was almost absent in RMUG-L cells. The results were consistent with those obtained in our previous study. We therefore chose SKOV-3 and RMUG-L cells for use in additional experiments. The staining intensity of phosphorylated EGFR, following 10 min of 100 nM PAF treatment, was much higher in SKOV-3 cells than in control cells, while there was no obvious change between the PAF treatment group and the control group in RMUG-L cells (Figure 2C and D). Furthermore, in SKOV-3 cells, the activation of EGFR through PAF was inhibited with increasing concentrations of WEB2086 [32], a specific small-molecular inhibitor of PAFR (Figure 2E). These data indicate the PAFR is required for EGFR transactivation in ovarian cancer cells.

### PAF activates ERK1/2 phosphorylation via both EGFR-dependent and EGFR-independent pathways

**Figure 3:**
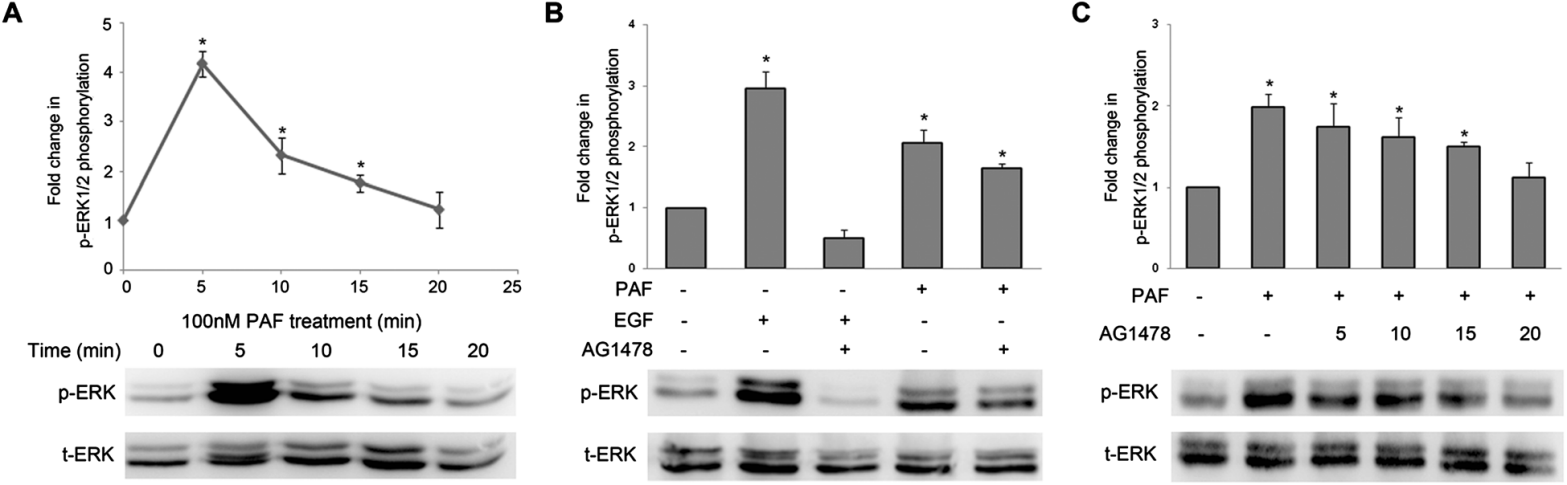
**PAF activates ERK1/2 signaling via both EGFR-dependent and EGFR-independent pathways. (A)** SKOV-3 cells were treated with 100 nM PAF for 0, 5, 10, 15, or 20 min. Following PAF treatment, the cells were lysed, and the lysates were evaluated by Western blotting. Data were normalized to total ERK protein expression and are expressed as fold-change (average ± S.E.M.) in phospho-ERK compared to vehicle-treated cells. Representative blots for phosphor/total-ERK are shown. **(B)** SKOV-3 cells were treated with AG1478 (20 μM) for 1 hour before stimulation with PAF (100 nM) or EGF (5 ng/ml) for 5 min. Cells were harvested and subjected to Western blot. **(C)** SKOV-3 cells were pretreated for 1 h with increasing concentrations of the EGFR inhibitor AG1478. Cells were then stimulated with PAF (100 nM) for 5 min before they were harvested and subjected to Western blot. The data shown are representative of at least three independent experiments. Data were analyzed by Student’s *t*-test. * *p* < 0.05.

ERK1/2 are kinases that promote survival and proliferation [33], and we have previously shown that ERK1/2 can be acutely activated through PAF and EGF receptor signaling pathways. Next, we examined the role of EGFR in the PAF-mediated activation of ERK1/2. As shown in Figure 3A, SKOV-3 cells were exposed with PAF (100 nM), and the stimulation evoked ERK1/2 phosphorylation in a time-dependent manner, with maximal activation at 5 min and a subsequent reduction to baseline after 20 min. We pretreated serum-starved SKOV-3 cells with the EGFR-specific small-molecule inhibitor AG1478 (20 μM), and the results showed that AG1478 only slightly inhibited the PAF-induced phosphorylation of ERK1/2 (Figure 3B). In addition, as shown in Figure 3C, increasing concentrations of AG1478 did not effectively inhibit the PAF-induced ERK1/2 activation. These results indicate that ERK1/2 might be phosphorylated through PAF via EGFR-dependent and EGFR-independent mechanisms in ovarian cancer cells.

### Involvement of calcium, but not PKC, in the PAF-induced transactivation of EGFR

**Figure 4:**
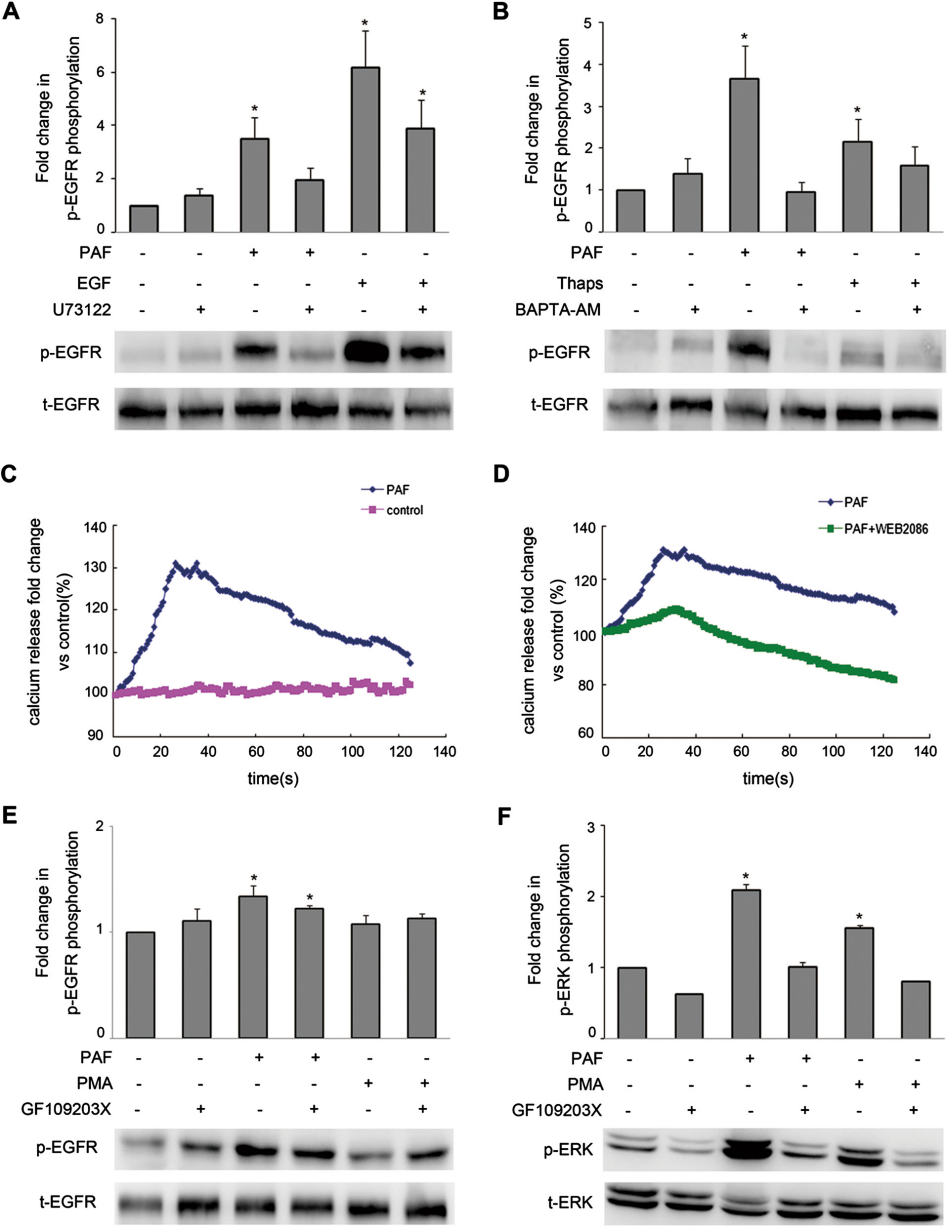
**Role of Ca** ^**2+**^ **and PKC in responses to PAF in SKOV-3 cells. (A)** SKOV-3 cells were pretreated for 1 h with the PLCβ inhibitor U73122 (20 μM) before stimulation with PAF (100 nM) or EGF (5 ng/ml) for 5 min. **(B)** SKOV-3 cells were pretreated for 1 h with the Ca^2+^ chelator BAPTA-AM (20 μM) before stimulation with PAF (100 nM) or thapsigargin (1 μM) for 5 min. **(C)** SKOV-3 cells were loaded with the calcium probe Fura-2/AM followed by stimulation with 100 nM PAF. **(D)** SKOV-3 cells were loaded with the calcium probe Fura-2/AM followed by stimulation with 100 nM PAF in the presence or absence of WEB2086, calcium mobilization was assayed by monitoring the change in Fura-2/AM fluorescence. **(E and F)** SKOV-3 cells were pretreated for 1 h with the PKC inhibitor GF109203X (5 μM) before stimulation with PAF (100 nM) or PMA (1 μM) for 5 min. Cells were then harvested and subjected to Western blot analysis. The data shown are representative of at least three independent experiments. Data were analyzed by Student’s *t*-test. * *p* < 0.05.

The available evidence indicates that G-protein coupled receptors (GPCR) mediate the activation of phospholipase C-β (PLCβ) leading to the formation of inositol trisphosphate (InsP_3_) and diacylglycerol (DAG) [34]. DAG directly activates classical types of PKCs, and IP3 activates intracellular Ca^2+^ [35]. Therefore, we then examined the potential involvement of PLCβ in the transactivation of EGFR using the PLC inhibitor U73122 (20 μM). SKOV-3 cancer cells were pretreated with 20 μM of U73122 for 1 hour, followed by PAF or EGF stimulation for 5 min. When cells were treated with PAF, U73122 significantly inhibited EGFR activation, while after treatment with EGF, U73122 only slightly affected EGFR activation (Figure 4A).

We next tried to determine which pathways downstream of PLCβ mediate the PAF-induced transactivation of EGFR. We observed the effects of the intracellular calcium chelator BAPTA-AM and thapsigargin, which increases intracellular calcium levels through the inhibition of the sarco/endoplasmic reticulum Ca^2+^-ATPase’ (SERCA) pump, on the PAF-induced phosphorylation of EGFR in SKOV-3 cells. The cells were pretreated with BAPTA-AM for 1 hour prior to PAF or thapsigargin stimulation for 5 min. As shown in Figure 4B, PAF and thapsigargin induced EGFR phosphorylation, while BAPTA-AM abrogated EGFR activation. Furthermore, we measured the effects of PAF and the PAF-receptor antagonist WEB2086 on intracellular calcium mobilization. As shown in Figure 4C and D, stimulation with PAF (100 nM) elicited a rapid increase in intracellular Ca^2+^ mobilization in SKOV-3 cells, and the PAF-induced Ca^2+^ mobilization could be inhibited through pretreatment with WEB2086 for 1 hour.

To determine whether PKC is a downstream pathways of PLCβ involving in EGFR transactivation mediated through PAF, SKOV-3 cells were pretreated with 20 μM of the PKC inhibitor GF109203X for 1 hour, followed by treatment with PAF or PMA, a PKC activator, for 5 min. As shown in Figure 4E, little EGFR inhibition was observed when SKOV-3 cells were treated with GF109203X; however, treatment with GF109203X resulted in dramatic decreases in ERK1/2 activation followed by stimulation with PAF or PMA (Figure 4F). These results suggest that Ca^2+^, rather than PKC, mediated the PAF-induced transactivation of the EGFR via PLCβ activation.

### Role of Src and metalloproteinases in the transactivation of EGFR

**Figure 5:**
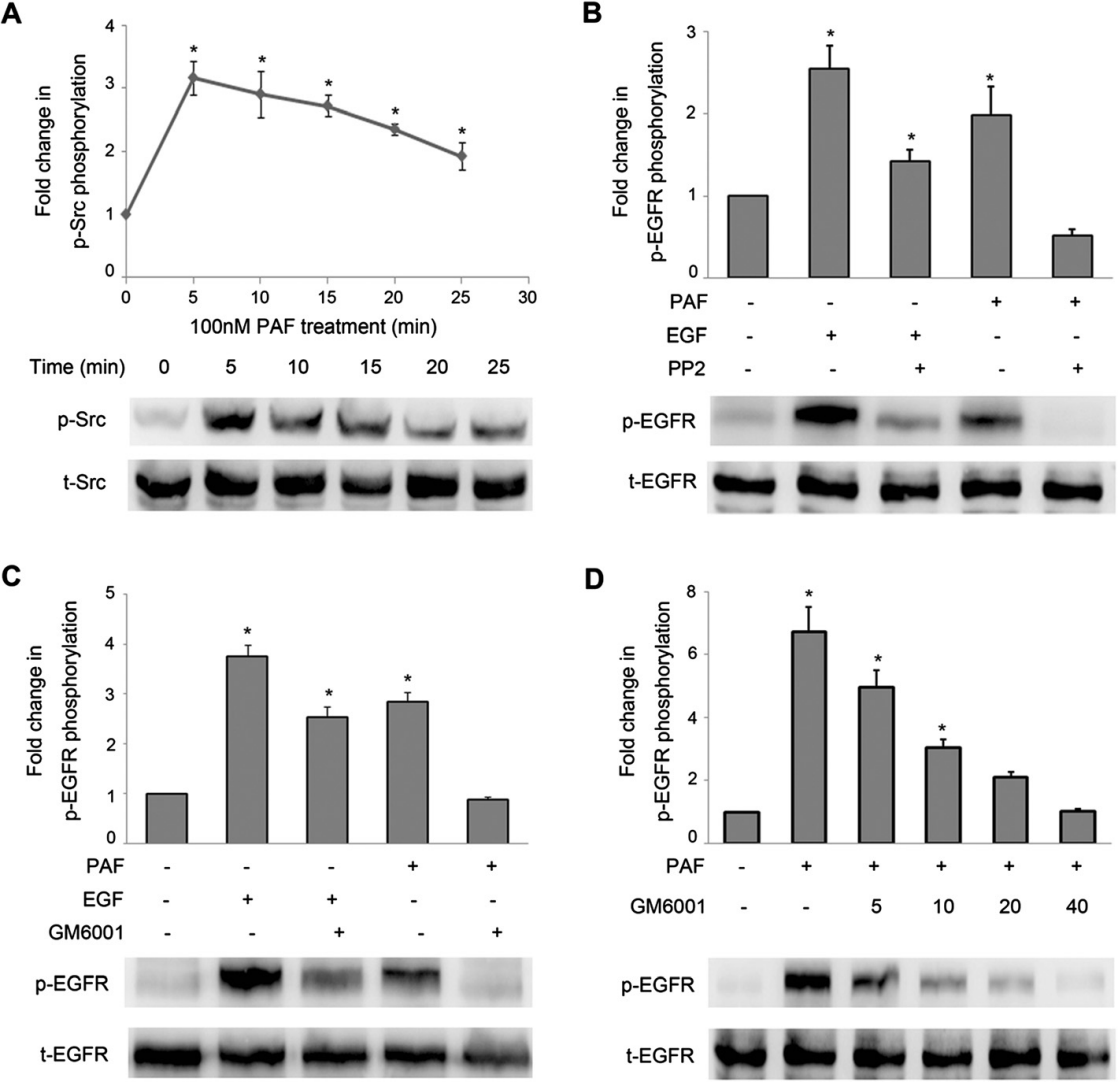
**Effect of Src and MMP inhibitors on EGFR phosphorylation. (A)** SKOV-3 cells were treated with 100 nM PAF for 0, 5, 10, 15, 20, or 25 min. Following PAF treatment, cells were lysed and lysates were evaluated by Western blotting. Data were normalized to total Src protein expression and are expressed as the fold change (average ± S.E.M.) in phospho-Src compared to vehicle-treated cells. Representative blots for phosphor/total-EGFR are shown. **(B)** SKOV-3 cells were treated with the Src inhibitor PP2 (20 μM) for 1 hour before stimulation with PAF (100 nM) or EGF (5 ng/ml) for 5 min. Cells were harvested and subjected to Western blot. **(C)** SKOV-3 cells were pretreated for 1 h with the metalloproteinase inhibitor GM6001 (20 μM). Cells were then stimulated with either PAF (100 nM) or EGF (5 ng/ml) for 5 min before they were harvested and immunoblotting. **(D)** SKOV-3 cells were pretreated for 1 h with increasing concentrations of the metalloproteinase inhibitor GM6001 before they were harvested and subjected to Western blot. The data shown are representative of at least three independent experiments. Data were analyzed by Student’s *t*-test. * *p* < 0.05.

To further elucidate the mechanisms underlying the induction of EGFR transactivation through PAF, we investigated the role of Src kinases. In SKOV-3 cells, PAF stimulation led to the activation of Src kinases in a time-dependent manner, with maximal activation at 5 min and a subsequent reduction to baseline after 25 min (Figure 5A). As shown in Figure 5B, pretreatment with the Src inhibitor PP2 (20 μM) abolished the PAF-induced phosphorylation of EGFR, but, had little effect on the phosphorylation of EGFR elicited through EGF. These results suggest the involvement of a Src family kinase in the PAF-induced transactivation of EGFR in SKOV-3 cells.

Previous evidence has implicated proteinases of the A-disintegrin-and metalloproteinase (ADAM) family in EGFR transactivation through GPCRs in various cells [36]. To examine the role of ADAMs in PAF-induced EGFR transactivation in SKOV-3 cells, we pretreated the cells with GM6001, a broad-spectrum metalloproteinase inhibitor. As shown in Figure 5C, this pretreatment resulted in the inhibition of PAF-induced EGFR phosphorylation, while the EGF-induced phosphorylation of these proteins was not affected. In addition, we pretreated cells with increasing concentrations of GM6001 and observed that GM6001 inhibited the EGFR phosphorylation in a dose-dependent manner (Figure 5D). These results indicate that transactivation is dependent on mechanisms involving the ADAM-mediated release of EGFR ligands.

### PAF-induced EGFR transactivation occurs via MMP-mediated release of HB-EGF

**Figure 6:**
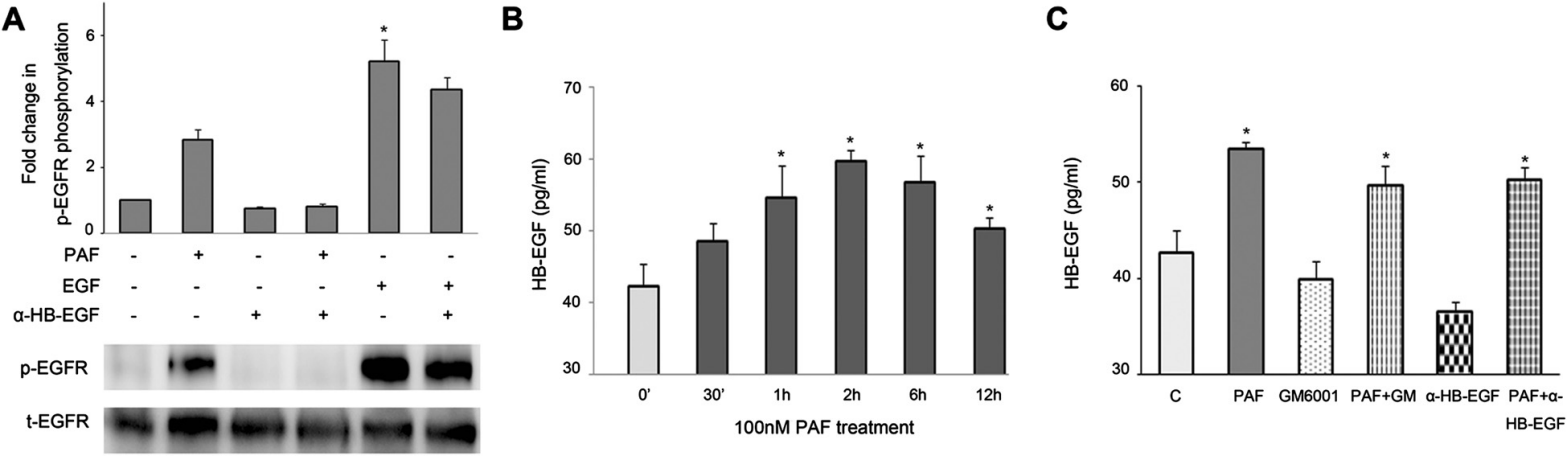
**Release of HB-EGF contributes to PAF-mediated EGFR transactivation. (A)** SKOV-3 cells were treated with the HB-EGF neutralizing antibody (50 μg/ml) for 1 hour before stimulation with PAF (100 nM) or EGF (5 ng/ml) for 5 min, cells were lysed and lysates were evaluated by Western blotting. Data were normalized to total EGFR protein expression and are expressed as the fold change (average ± S.E.M.) in phospho-EGFR compared to vehicle-treated cells. Representative blots for phosphor/total-EGFR are shown. **(B)** SKOV-3 cells were treated with PAF (100 nM) for the indicated time (30 min to 12 h) while measuring HB-EGF production. **(C)** SKOV-3 cells were serum starved and then pretreated with 20 μM GM6001 or HB-EGF neutralizing antibody (50 μg/ml) for 1 h. cells were then stimulated with 100 nM PAF for 2 h. The medium was harvested, and the amount of HB-EGF was measured. Bars represent the average of triplicates ± S.D.; * p < 0.05 indicate a statistically significant difference compared to the untreated control.

EGFR can be activated through several ligands. EGFR ligands bind to the extracellular domain of EGFR, triggering signaling downstream cascades, including MAPK [37-39]. As shown in Figure 6A, SKOV-3 cells were pretreated with the HB-EGF neutralizing antibody for 1 hour, followed by PAF or EGF stimulation for 5 min, and this treatment inhibited PAF-induced EGFR phosphorylation, while the EGF-induced phosphorylation of these proteins was hardly affected. As shown in Figure 6B, in SKOV-3 cells, HB-EGF production increased after 30 min of stimulation with PAF (100 nM) and continued to increase, reaching a maximum after 2 hours. Longer PAF stimulation caused no significant additional increase in HB-EGF release over that obtained at 2 hours. In addition, we examined the effects of the metalloproteinase inhibitor GM6001 on HB-EGF production in SKOV-3 cells. The cells were pretreated with GM6001 and the HB-EGF neutralizing antibody for 1 hour prior to PAF stimulation for 2 hours. Using an enzyme-linked immunosorbent assay, we analyzed HB-EGF production in the medium of SKOV-3 cells. HB-EGF levels increased in the medium after PAF stimulation and GM6001 treatment inhibited PAF-induced HB-EGF production (Figure 6C). These results indicated that the ADAM-mediated release of HB-EGF is involved in the PAF-induced transactivation of EGFR.

## Discussion

**Figure 7:**
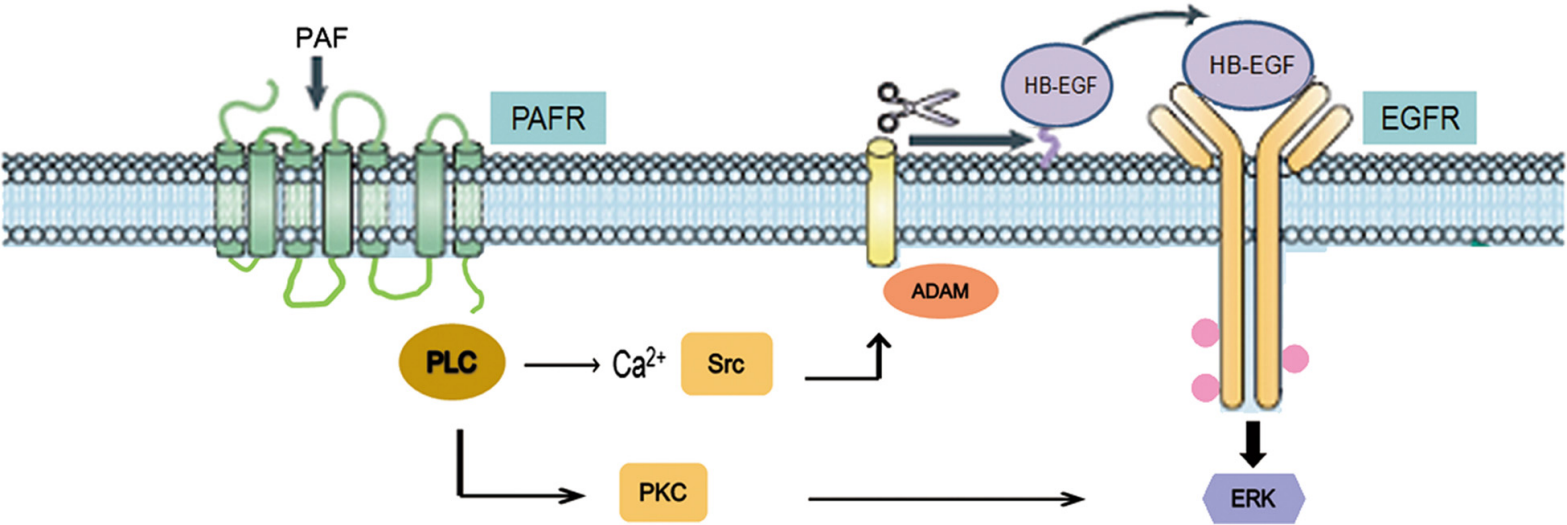
**Proposed model of the mechanisms involved in PAF-mediated EGFR transactivation in SKOV-3 ovarian cancer cells.** In response to PAF, activated PAFR triggers the EGFR/ERK transactivation pathway via PLCβ, intracellular Ca^2+^, Src and the ADAM-mediated release of EGFR ligand HB-EGF. PAF also triggers the PKC pathway to couple to ERK phosphorylation.

In the present study, we demonstrated that PAFR is capable of stimulating the activation of EGFR and ERK1/2 in ovarian cancer cells endogenously expressing PAFR. PAFR-mediated ERK1/2 activation appeared to be secondary to EGFR stimulation, as the EGFR tyrosine kinase inhibitor AG1478 blocked PAF-induced ERK1/2 activation. However, the ERK1/2 activation induced through PAF is not entirely dependent on EGFR stimulation. As illustrated in the proposed model (Figure 7), the transactivation of the EGFR through PAF involves the PAFR, the activation of PLCβ with downstream signaling through Ca^2+^ mobilization, the tyrosine-protein kinase Src, matrix metalloproteinase- dependent cleavage and the secretion of HB-EGF which activates the EGFR and subsequently phosphorylates ERK1/2. The transactivation of EGFR through other G-protein coupled receptors (GPCR) is an important signaling pathway for the induction of both mitogenic and motogenic effects, suggesting a mechanism for the proliferative effects of PAF in ovarian cancer cells.

As a member of G-protein coupled receptors, PAFR is involved in many biological functions, including inflammation, cell growth, chemotaxis and oncogenesis. Several recent reports provide evidences linking inflammation and cancer promotion [40,41]. Inflammatory moleculars, such as PAF and LPA, in the tumor microenvironment bind GPCRs and induce mitogenesis and invasion in several cancers, including ovarian cancer [42,43]. The GPCR-mediated transactivation of EGFR leads to the downstream activation of ERK1/2. Depending on the cell type and the GPCR ligand, ERK1/2 might be activated via EGFR-dependent and/or EGFR-independent mechanisms [44,45]. In the present study, we observed that PAF-induced ERK1/2 phosphorylation can be inhibited with the EGFR tyrosine kinase inhibitor AG1478, but this inhibition was slightly. In addition, increasing concentrations of AG1478, did not lead to the obvious inhibition of ERK1/2 phosphorylation. These results suggest that there might be other mechanisms of PAF-induced ERK1/2 activation.

When G-protein coupled receptors were activated, downstream PLCβ could regulate cellular functions through two distinct pathways involving the DAG-mediated activation of PKC and the InsP3-induced release and elevation of cytosolic Ca^2+^, respectively. Our findings suggest that in the SKOV-3 cells, PAF-induced EGFR transactivation was mediated through Ca^2+^, as the PKC activator PMA did not mimic this effect and the PKC blocker GF109203X did not inhibit this effect. However, thapsigargin, which elevates intracellular Ca^2+^, mimicked the PAF effect, and the Ca^2+^ chelator BAPTA-AM blocked the transactivation of EGFR phosphorylation. However, the PKC activator PMA activated the phosphorylation of ERK, and the phosphorylation of ERK was inhibited by PKC inhibitor GF109203X, indicating that PAF activates ERK, the downstream target of EGFR, via PLCβ-dependent PKC and MMP-mediated EGFR pathways to promote ovarian cancer progression.

In different types of cells, both ligand-dependent and ligand-independent mechanisms mediate EGFR transactivation [46]. Ligand-dependent mechanisms involve the release of EGFR agonists through the cleavage and shedding of membrane-associated precursors by proteinases of the ADAM family [47]. Ligand-independent mechanisms have been reported to involve intracellular molecules, including Src family kinases and Pyk2 [48,49]. In the SKOV-3 cells, we observed that Src inhibitors abolished the PAF-induced phosphorylation of the EGFR. In contrast, these inhibitors only slightly affected the response to EGF, suggesting a role for Src in the transactivation of EGFR in SKOV-3 cells. We also observed the involvement of HB-EGF shedding in the transactivation of EGFR after PAF stimulation, as pretreatment with the metalloproteinase inhibitor GM6001 almost completely prevented the PAF-induced, but not EGF-induced, phosphorylation of EGFR in these cells. HB-EGF is released after PAF treatment, and increased HB-EGF concentrations are blocked through the metalloproteinase inhibitor GM6001. These results indicate that in the SKOV-3 cells, Src is involved in the activation of ADAMs, rather than directly stimulating the EGFR, thus combining these two mechanisms.

EGFR is expressed at high levels in ovarian cancer, where signaling through EGFR contributes to cell survival, proliferation and invasion. The inhibition of EGFR alone, has resulted in limited antitumor effects when tested as a monotherapy in clinical trials. In addition to receptor tyrosine kinase, PAFR regulates the responsiveness of cancer cells to the ligand PAF. It has become increasingly evident that the crosstalk between PAFR and EGFR confer an aggressive tumorigenic phenotype in cancer cells. In the present study, we have shown that the activation of both PAFR and EGFR induces the phosphorylation of the MAPK pathway, and PAF mediates the transactivation of EGFR. Indeed, combined treatment with an EGFR tyrosine kinase inhibitor and the PAFR antagonist resulted in additive antitumor effects. We have shown that PAF stimulates ERK1/2 through different mechanisms, acting by PAFR-mediated EGFR transactivation in ovarian cancer cells. This evidence further confirms the diversity of intracellular crosstalk and underscores the importance of investigating these mechanisms to better understand signaling in cancer cells.

## Conclusions

In the present study, we demonstrated that PAF transactivates EGFR through PAFR in both time- and dose-dependent manners in the SKOV-3 ovarian cancer cells. EGFR transactivation was dependent on the PAF-receptor, PLCβ, Ca^2+^, Src and the ADAM-mediated release of HB-EGF. These results support the development of therapeutic strategies involving the combined EGFR and PAFR targeting in ovarian cancer.

## Abbreviations

PAF: Platelet-activating factor
PAFR: PAF-receptor
EGF: Epidermal growth factor
EGFR: EGF-receptor
HB-EGF: Heparin-binding epidermal growth factor
GPCR: G-protein coupled receptor (GPCR) family
PLCβ: Phospholipase C-β
ERK: Extracellular-regulated protein kinase
InsP_3_: Inositol trisphosphat
DAG: Diacylglycerol
ADAM: A disintegrin and metalloproteinase
MMP: Metalloproteinase
AG1478: An EGFR-specific tyrosine kinase inhibitor
WEB2086: A specific PAFR antagonist
